# Rictor/mTORC2 involves mitochondrial function in ES cells derived cardiomyocytes via mitochondrial Connexin 43

**DOI:** 10.1038/s41401-020-00591-3

**Published:** 2021-02-05

**Authors:** Jia-dan Wang, Ying Shao, Dan Liu, Nuo-ya Liu, Dan-yan Zhu

**Affiliations:** 1 Institute of Pharmacology and Toxicology, Zhejiang University, Hangzhou, 310058 China; 2 Xiaoshan Traditional Chinese Medical Hospital, Hangzhou, 311201 China

**Keywords:** Rictor/mTORC2, embryonic stem cell, mitochondria, Connexin43, cardiomyocyte differentiation

## Abstract

Rictor is a key component of the mammalian target of rapamycin complex 2 (mTORC2) and is required for Akt phosphorylation (Ser473). Our previous study shows that knockdown of Rictor prevents cardiomyocyte differentiation from mouse embryonic stem (ES) cells and induces abnormal electrophysiology of ES cell-derived cardiomyocytes (ESC-CMs). Besides, knockdown of Rictor causes down-expression of connexin 43 (Cx43), the predominant gap junction protein, that is located in both the sarcolemma and mitochondria in cardiomyocytes. Mitochondrial Cx43 (mtCx43) plays a crucial role in mitochondrial function. In this study, we used the model of cardiomyocyte differentiation from mouse ES cells to elucidate the mechanisms for the mitochondrial damage in ESC-CMs after knockdown of Rictor. We showed swollen and ruptured mitochondria were observed after knockdown of Rictor under transmission electron microscope. ATP production and mitochondrial transmembrane potential were significantly decreased in Rictor-knockdown cells. Furthermore, knockdown of Rictor inhibited the activities of mitochondrial respiratory chain complex. The above-mentioned changes were linked to inhibiting the translocation of Cx43 into mitochondria by knockdown of Rictor. We revealed that knockdown of Rictor inactivated the mTOR/Akt signalling pathway and subsequently decreased HDAC6 expression, resulted in Hsp90 hyper-acetylation caused by HDAC6 inhibition, thus, inhibited the formation of Hsp90-Cx43-TOM20 complex. In conclusion, the mitochondrial Cx43 participates in shRNA-*Rictor*-induced mitochondrial function damage in the ESC-CMs.

## Introduction

The heart is the first functional organ formed during embryonic development [1]. And a high mitochondrial density is crucial to meet the energy demands during embryo cardiac development. The mammalian target of rapamycin complex 2 (mTORC2) pathway was shown to participate in protecting the heart from ischaemic injury, cell proliferation and differentiation [2, 3]. Furthermore, mTORC2 regulates mitochondrial reactive oxygen species (ROS) and mitochondrial respiration [4]. Rictor is a core component of mTORC2 [5]. Embryos that lacked Rictor exhibited growth arrest and died at E11.5 [6]. In addition, Rictor deletion in embryonic heart tissue was revealed to contribute to heart defects [7].

Mouse embryonic stem (ES) cells differentiated into cardiomyocytes in vitro serve as a suitable model to find new targets for regulating cardiomyocyte formation and to study embryonic cardiac development [8]. Recent studies have demonstrated that mitochondria affect cardiomyocyte differentiation via altering states of mitochondrial permeability transition pore opening [1, 9], mitochondrial fusion [10], mitochondrial DNA transcription factors [11], and mitochondrial reactive ROS [12]. Our previous study showed that knockdown of Rictor inhibited mouse ES cell differentiation into cardiomyocytes. The differentiated cardiomyocytes exhibited irregular myofilaments and disordered electrophysiological activity [13]. In addition, we found that the structure of the mitochondria-endoplasmic reticulum membrane was damaged after knockdown of Rictor, which inhibited the release of calcium from the endoplasmic reticulum to mitochondria [14]. Thus, we speculated that Rictor affects the cardiomyocyte differentiation of ES cells by regulating mitochondrial function.

Connexin 43 (Cx43), the predominant gap junction protein, is mainly localized in the sarcolemma but is also found in the mitochondria and binds heat shock protein 90 (Hsp90) and Tom20 [15–17]. Mitochondrial Cx43 (mtCx43) forms a hemichannel in the mitochondrial inner membrane, regulating mitochondrial potassium uptake [18], ROS generation [19], and energy metabolism [20]. Mitochondrial Cx43 hemichannels were shown to contribute to mitochondrial calcium homoeostasis and cell injury/death in the heart [21]. Studies have shown that mtCx43 specifically affects the activity of respiratory chain enzyme complex I [22]. Our previous study found that knockdown of Rictor led to decreased expression of Cx43 in gap junctions in ES cell-derived cardiomyocytes (ESC-CMs) [13]. We speculated that mtCx43 was involved in regulating mitochondrial function by Rictor, further influencing energy metabolism, leading to repression of the cardiomyocyte differentiation of ES cells.

The activity of Akt can be regulated by Rictor through Akt phosphorylation (Ser473) [23]. It has been reported that HDAC6 is regulated by the PTEN/Akt/mTOR pathway [24]. Furthermore, the acetylation of Hsp90, mediated by HDAC6, has been shown to be involved in the mitochondrial transport of proteins [25]. In addition, Cx43 transport to the mitochondrial inner membrane was found to be dependent on the Hsp90-TOM20 system. However, whether knockdown of Rictor contributed to mtCx43 expression by downregulating HDAC6 and the mechanism of Rictor-regulated mitochondrial function in ESC-CMs remained unclear.

In this study, a model of cardiomyocyte differentiation in mouse ES cells was employed to investigate the mechanism of mitochondrial damage in ESC-CMs after the knockdown of Rictor.

## Materials and methods

### Cell culture and cardiomyocyte differentiation

D3 mouse ES cells (American Type Culture Collection, VA, USA) were maintained as previously described [26, 27]. Briefly, mouse ES cells (~900) were cultured in a 30-µL hanging droplet for 3 d in differentiation medium containing DMEM (Gibco, Life Technologies, WA, USA) with 20% FBS (Gibco), 0.1 mmol·L^−1^ β-mercaptoethanol (Sigma-Aldrich, MO, USA), and 1% NEAAs (Gbico) to form embryoid bodies (EBs). On d 3, the EBs were transferred to Petri dishes and floated for an additional 2 d. Then, on d 5, the EBs were individually plated onto cell culture plates for an additional 3 d.

### Infection with short hairpin RNA (shRNA) targeting Rictor and overexpressing mtCx43

Lentiviruses containing *Rictor* shRNA and *control* shRNA and lentiviruses contain *mtCx43* were used to infect mouse ES cells. Lentiviruses contain *Rictor* or *control* shRNA was used in the group of shRNA-*Con* and shRNA-*Rictor* respectively. In the group of shRNA-*Rictor*+mtCx43, lentiviruses contain *mtCx43* and lentiviruses contain *Rictor* shRNA were used together in order to knockdown of Rictor and over express mtCx43. shRNA-*Rictor* and shRNA-*Con* were ordered from Genepharma Company (Shanghai, China). Mouse *mtCx43* labeled with RFP was ordered from Hanheng Biotechnology Company (Shanghai, China).

shRNA-*Rictor*: 5‵-GCCAGTAAGATGGGAATCATT-3‵,

shRNA-*Con*: 5‵-TTCTCCGAACGTGTCACGTTC-3‵,

and sequence target mitochondria: 5‵-ATGTCCGTCCTGACGCCGCTGCTGCTGCGGGGCTTGACAGGCTCGGCCCGGCGGCTCCCAGTGCCGCGCGCCAAGATCCATTCGTT-3‵.

Briefly, 1 × 10^5^ mouse ES cells per well were seeded into 12-well plates. The cells were infected with an aliquot of lentivirus to achieve a multiplicity of infection of 50 PFU/cell 4 h later. After infection for 24 h, cells were harvested for EB formation.

### Isolation of ESC-CMs

On d 5 + 3, the beating areas of the EBs were dissociated by gentle pipetting with a glass pipette with an internal diameter of 200–300 μm. Then, the cell clusters were digested into individual cells with Accutase enzyme solution (Life Technologies) for 30 min at 37 °C. The isolated cells were collected for further analyses [28].

### Western blot analysis

Cell lysates were prepared in Western blot lysis buffer (Beyotime, Shanghai China). Mitochondrial/cytoplasmic proteins were obtained from samples with a mitochondrial protein extraction kit (Beyotime). Samples were coimmunoprecipitated using Protein A + G agarose beads (Beyotime) according to the manufacturer’s instructions. Western blotting was performed as previously reported [26, 27]. Antibodies against thefollowing proteins were used: GAPDH, α-actinin (Sigma Aldrich), Akt1/2/3 (Ser 473), Akt1/2/3, Rictor,  VDAC1 (Santa Cruz, TX, USA), cytochrome *c*, Oct4, mTOR, p-mTOR (Ser2481), HDAC6, TOM20 (Cell Signalling Technology, MA, USA), c-TNT, Connexin 43, Rictor, Hsp90, SIN1, G protein beta subunit like,  and acetyl-lysine (Abcam, MA, USA). The membranes were incubated with horseradish peroxidase (HRP)-conjugated antibodies (Lianke, Hangzhou, China). All data analyses were carried out by using ImageJ software.

### Flow cytometry analysis

EBs obtained on d 5 + 3 of differentiation were harvested and digested into single cells with Accutase enzyme solution (Life Technologies). Cells were fixed with 4% paraformaldehyde for 1 h and then blocked with 5% bovine serum albumin (BSA, Sigma Aldrich) for another 1 h at room temperature. After that, the cells were incubated with monoclonal anti-α-actinin antibody (Sigma Aldrich, 1:400) or monoclonal anti-c-TNT antibody (Abcam, 1:400) overnight. After being washed with PBS three times, the cells were incubated with DyLight 488-conjugated anti-mouse IgG (1:400) at 4 °C for 1 h. Then, a total of 1 × 10^4^ cells were suspended in 0.5 mL of 1% BSA and analyzed by FACScan flow cytometry (Becton Dickinson, NJ, USA) [26, 29].

### Measurement of mitochondrial membrane potential (ΔΨm)

The ΔΨm of ESC-CMs was measured as previously described [26] following the manufacturer’s instructions. ESC-CMs on d 5 + 3 were plated on 48-well plates at a density of 3 × 10^4^ cells per well. After 48 h, the cells were incubated with 2 μg·mL^−1^ JC-1 dye (Beyotime) at 37 °C in the dark for 30 min. Then, the cells were washed with washing buffer, and images were obtained with a Leica DMI3000B microscope (Leica Microsystems, Wetzlar, Germany).

### Cellular ATP assay

ESC-CMs on d 5 + 3 were assessed with an ATP bioluminescence assay kit (S0026, Beyotime) following the manufacturer’s instructions. All the data were standardized to the control group.

### Immunofluorescence analysis

Immunofluorescence analysis of ESC-CMs on d 5 + 3 was performed as previously described [26, 27]. Cells were incubated with MitoTracker or ER-Tracker (Life Technologies) for 30 min in the dark and then fixed with 4% paraformaldehyde for 30 min. The cells were then blocked with 5% BSA for 1 h at room temperature. After that, the cells were incubated with antibody against Cx43 (Abcam, 1:400) or Rictor (Abcam, 1:400) at 4 °C overnight. Next, the cells were washed with PBS three times and then incubated with Alexa Fluor 488-conjugated anti-rabbit IgG (Lianke, 1:400) or DyLight 650-conjugated anti-rabbit IgG (Invitrogen, CA, USA, 1:400) antibody at 4 °C for 2 h. The cells were observed under an Olympus FV3000 confocal microscope (Olympus, Hertfordshire, UK).

### Transmission electron microscopy

ESC-CMs on d 5 + 3 were fixed with 2.5% glutaraldehyde at 4 °C for 2 h and 1% osmium tetroxide for 1.5 h. Then, the cells were dehydrated with gradient ethanol (30%, 50%, 70%, 80%, 90% and 100%) and pure acetone. The cells were embedded in pure embedding agent and sectioned at a thickness of 70–90 nm. Then, the samples were dyed with  lead citrate and uranyl acetate solutions and observed under a transmission electron microscope [26].

### Respiratory chain enzyme assays

The activities of individual enzymes in complexes I, III, IV and V were measured following the manufacturer’s instructions. Complex I activity was measured by monitoring NADH oxidation at 340 nm. Complex III and IV activities were measured by monitoring the reduction andoxidation, respectively, of cytochrome *c* at 550 nm. Citrate synthase was assayed by monitoring 5,5’-dithiobis (2-nitrobenzoid) reduction at 412 nm.

### Statistics analysis

Data are expressed as the mean values ± standard deviations. At least 3 independent experiments were performed as replicates. Statistical analyses were performed by *t* test when two groups were compared. A value of *P* < 0.05 indicated a significant difference.

## Results

### Knockdown of Rictor impaired mitochondrial structure and function in ESC-CMs

**Fig. 1:**
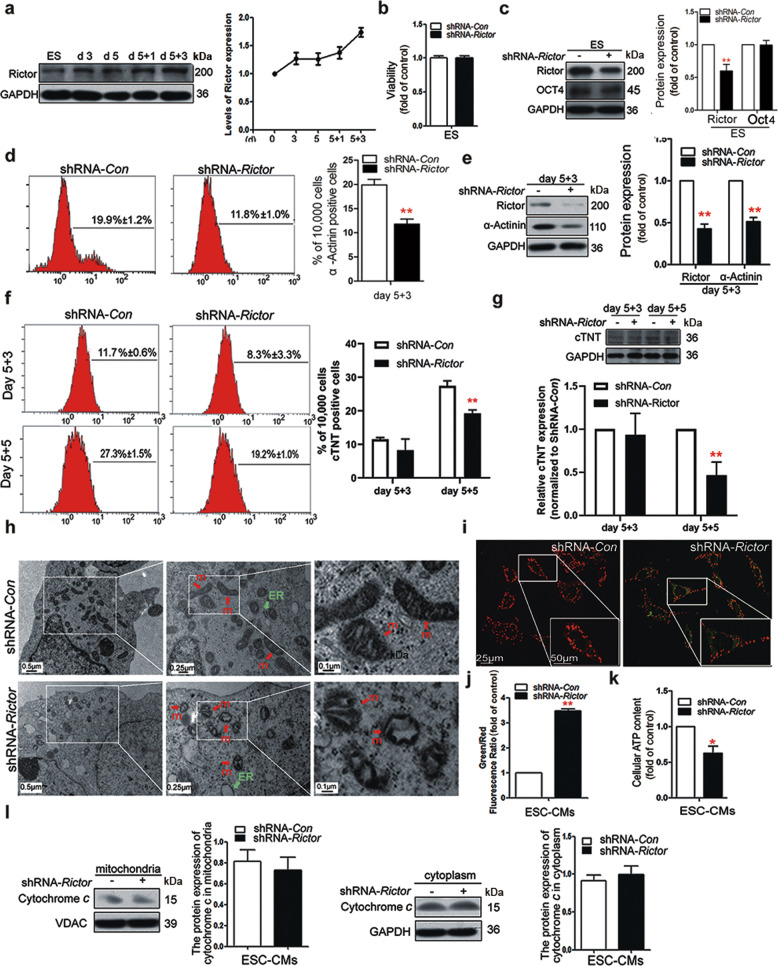
Knockdown of Rictor inhibited cardiomyocyte differentiation and impaired the mitochondrial structure and function in ESC-CMs. **a** The expression of Rictor/mTORC2 during cardiomyocyte differentiation. **b** The cell viability of ES cells after transfection with shRNA lentivirus. **c** The expression of Rictor and Oct4 after transfection with shRNA lentivirus. **d** Proportions of α-actinin-positive cells in the shRNA-*Con* and shRNA-*Rictor* groups on d 5 + 3 determined by flow cytometry. **e** The protein expression of α-actinin was evaluated in EBs on d 5 + 3. **f** The proportions of c-TNT-positive cells in the shRNA-*Con* and shRNA-*Rictor* groups on d 5+3 and d 5+5, determined by flow cytometry. **g** The protein expression of c-TNT was evaluated in EBs on d 5 + 3 and d 5 + 5. **h** The ultrastructure of ESC-CMs after Rictor knockdown. (m: mitochondrion, ER: endoplasmic reticulum). **i** The ΔΨm of ESC-CMs was evaluated with JC-1 staining. **j** The ΔΨm of ESC-CMs was further assessed by flow cytometry. **k** Intracellular ATP production by ESC-CMs. **l** The expression levels of cytochrome *c* in the mitochondria and cytoplasm were no different between the two groups. *n* ≥ 3. *P < 0.05, **P < 0.01 vs shRNA-*Con*. Bars = 0.1 μm, 0.25 μm, 0.5 μm, 25 μm, and 50 μm.

The level of Rictor during the cardiomyocyte differentiation of mouse ES cells was upregulated, as shown by examination by Western blot analysis (Fig. 1a). After the transfection of shRNA-*Rictor* lentivirus, the cell viability and pluripotency of the ESCs were not different from those of the shRNA-*control* group (Fig. 1b, c). Knockdown of Rictor inhibited cardiomyocyte differentiation. After the knockdown of Rictor, the proportion of cells positive for α-actinin (a cardiomyocyte biomarker) on d 5 + 3 was significantly decreased to 11.8% ± 1.0% compared to that in the control group (19.9% ± 1.2%), as shown by flow cytometry analysis (Fig. 1d). The Western blot results also showed that the expression level of α-actinin was markedly reduced in Rictor-knockdown cells (Fig. 1e). In addition, the presence of Troponin T in cardiomyocytes (cTNT) typically indicates a relatively late phase of differentiation. We detected the expression of cTNT by Western blot analysis and flow cytometry. The results showed no significant difference in cTNT levels between the shRNA-*Con* and shRNA-*Rictor* groups on days 5 + 3, while on day 5 + 5, the expression of cTNT was decreased in the shRNA-*Rictor* group compared to the shRNA-*Con* group (Fig. 1f, g). These results demonstrated that knockdown of Rictor prevented cardiomyocyte differentiation from mouse ES cells. Cardiomyocytes with a purity of 83.4 %± 3.7% (Supplementary Fig. 1) were used to explore mitochondrial function in ESC-CMs. Transmission electron microscopy showed that the mitochondria in shRNA-*Rictor*-treated ESC-CMs appeared swollen and contained more vacuoles (Fig. 1h). We further examined the ΔΨm by JC-1 staining and found that ESC-CMs in the Rictor-knockdown group exhibited a lower ratio of red/green fluorescence intensity than those in the control group (Fig. 1i). Flow cytometry analysis also showed that the ratio of green (FL1-H)/red (FL2-H) fluorescence was significantly increased in Rictor-knockdown cells, indicating a low ΔΨm (Fig. 1j). The intracellular ATP level on d 5 + 3 was significantly decreased in shRNA-*Rictor* cells compared to the control group (Fig. 1k). To determine whether shRNA-*Rictor* transfection triggered apoptosis, the release of cytochrome *c* from the mitochondria was measured by Western blot analysis. The mitochondrial and cytoplasmic cytochrome *c* contents were not significantly different between the two groups (Fig. 1l).

### mtCx43 is involved in regulating mitochondrial function in ESC-CMs, as shown by shRNA-*Rictor* transfection

**Fig. 2:**
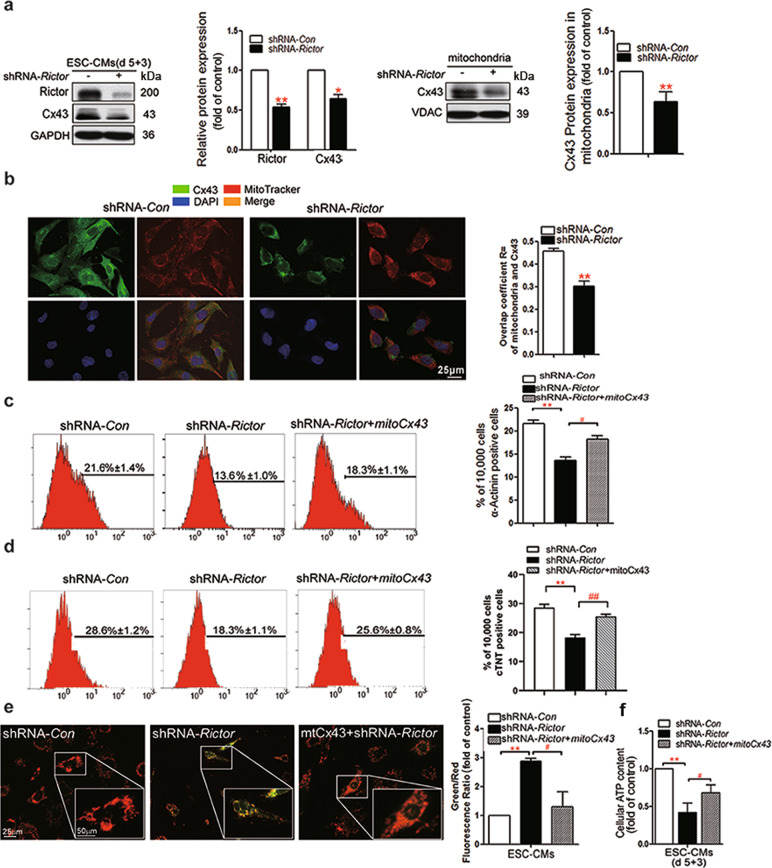
MtCx43 is involved in the shRNA-Rictor-induced damage to mitochondrial function. **a** The protein expression levels of Cx43 in the total cell lysate and mitochondria. **b** ESC-CMs were double-stained for Cx43 (green) and mitochondria (red). **c** The proportion of α-actinin-positive cells in EBs on d 5 + 3 was determined by flow cytometry analysis. **d** The proportions of c-TNT-positive cells in EBs on d 5 + 5 were determined by flow cytometry analysis. **e** The ΔΨm in ESC-CMs were detected by JC-1 staining and flow cytometry. **f** Intracellular ATP production was assessed in ESC-CMs at d 5 + 3. *n* ≥ 3. **P < 0.01 vs shRNA-*Con*; ^#^*P* < 0.05, ^##^*P* < 0.01 vs shRNA-*Rictor*. Bars = 25 μm and 50 μm.

The results of Western blot assays showed that total Cx43 and mtCx43 were significantly reduced in shRNA-*Rictor* cells (Fig. 2a). The ratio of Cx43 (green) and MitoTracker (red) (Fig. 2b) colocalization was significantly downregulated by shRNA-*Rictor* transfection compared with that in the control group. An increased cardiomyocyte differentiation efficiency was found in cells transfected with both shRNA-*Rictor* and *mtCx43*. The proportion of α-actinin-positive cells on d 5 + 3 was increased to 18.3% ± 1.1% compared with 13.6% ± 1.0% in the shRNA-*Rictor* group on d 5 + 3, as shown by flow cytometry analysis (Fig. 2c). In addition, flow cytometry analysis showed that the proportion of c-TNT-positive cells on d 5 + 5 was increased to 25.6% ± 0.8% compared with 18.3% ± 1.1% in the shRNA-*Rictor* group (Fig. 2d). To investigate whether Rictor regulates the function of mitochondria via mtCx43, we overexpressed mtCx43 in ES cells. Overexpression of mtCx43 mitigated the shRNA-*Rictor*-induced decrease in intracellular ATP production and ΔΨm (Fig. 2e, f).

### Rictor-mediated regulation of the mitochondrial respiratory chain via mtCx43

**Fig. 3:**
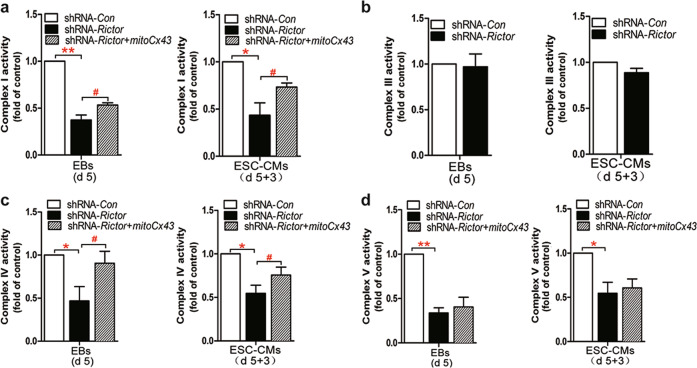
Rictor-mediated regulation of the mitochondrial respiratory chain via mtCx43. The activities of enzymes in complexes I (**a**), III (**b**), IV (**c**), and V (**d**) in Rictor-knockdown ESC-CMs were determined. *n* ≥ 3. *P < 0.05, **P < 0.01 vs shRNA-*Con*; ^#^*P* < 0.05 vs shRNA-*Rictor*.

Assays to detect the activities of respiratory chain enzymes showed that the activities of complex I, IV, and V were markedly reduced in shRNA-*Rictor* cells (Fig. 3a, c, d), while complex III activity was not significantly changed (Fig. 3b). Decreases in the activities of complexes I and IV were significantly reversed in ESC-CMs transfected with shRNA-*Rictor* and *mtCx43* compared with the shRNA-*Rictor* group (Fig. 3a, c). Overexpression of mtCx43 slightly attenuated the decrease in complex V activity caused by the knockdown of shRNA-*Rictor*, but this effect did not reach statistical significance (Fig. 3d).

### The mechanism by which Rictor regulates the translocation of Cx43 to mitochondria

**Fig. 4:**
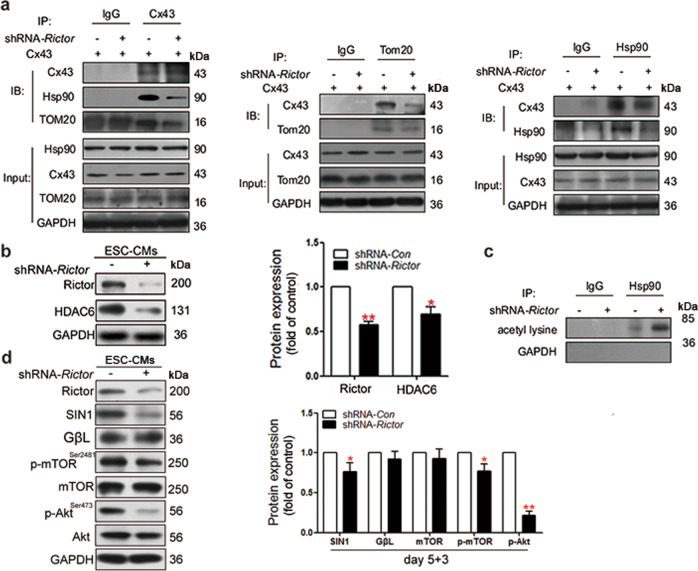
The mechanism by which Rictor regulates the translocation of Cx43 to mitochondria. **a** The interaction of Cx43 with the mitochondrial protein import system (Hsp90, TOM20) in ESC-CMs was decreased after knockdown of Rictor. **b** The protein expression level of HDAC6 in ESC-CMs. **c** Knockdown of Rictor increased the acetylation of Hsp90 in ESC-CMs. **d** Knockdown of Rictor decreased the expression of SIN1, p-Akt^Ser473^, and p-mTOR^Ser2481^ in ESC-CMs. *n* ≥ 3. *P < 0.05, **P < 0.01 vs shRNA-*Con*.

Cx43 is translocated to the inner mitochondrial membrane through the Hsp90-dependent TOM20 pathway [30]. Knockdown of Rictor led to a significant decrease in the interactions of Hsp90 and TOM20 with Cx43 in ESC-CMs (Fig. 4a). Decreased levels of p-HDAC6 and increased Hsp90 acetylation were observed in Rictor-knockdown cells compared to the control group (Fig. 4b, c). Furthermore, knockdown of Rictor destroyed the integrity and activity of mTORC2, as evidenced by diminished levels of p-mTOR^Ser2481^, SIN1 and p-Akt^Ser473^ (Fig. 4d).

## Discussion

mTORC2 was reported to play important roles in the development of embryonic and extraembryonic tissues. The formation of EBs from human amniotic fluid stem cells was found to depend on the activities of mTORC2 [31]. The mTORC2 consists of Rictor, mTOR, GβL and SIN1. Among these, Rictor is the core of the mTORC2 complex [32]. Embryos with Rictor knockdown showed growth arrest at E9.5, death at E13.5 and defective placental development [6]. Moreover, Rictor was confirmed to be crucial to embryonic heart development [7]. Similarly, in our study, we found that the protein expression of Rictor increased during the process of cardiomyocyte differentiation of mouse ES cells. Knockdown of Rictor significantly reduced cardiomyocyte differentiation efficiency, suggesting that Rictor/mTORC2 had a positive effect on cardiomyocyte differentiation.

In cardiomyocytes, the phosphorylation of Akt at Ser473 is modulated by the upstream regulator mTORC2 [7]. In addition, the PI3K-Akt-mTOR pathway regulates the metabolism and proliferation of cardiomyocytes. In our study, we found that knockdown of Rictor destroyed the integrity of mTORC2 and then decreased the phosphorylation of Akt at Ser473. mTORC2 modulates mitochondrial function via Akt, including its increase of ATP production and mitochondrial membrane potential [33]. Cardiac mitochondria play a crucial role in the maintenance of cellular bioenergetics and energy metabolism, as they provide ATP to meet the energy demand in cardiomyocyte differentiation and excitation-contraction coupling [34]. Further evidence has revealed that mtCx43 modulates mitochondrial function in different ways, such as its involvement in the homoeostasis of iron, calcium and potassium and reactive oxygen species generation [21]. In our study, we found that upon the knockdown of Rictor in ESC-CMs, the translocation of Cx43 into mitochondria was decreased, and the mitochondrial structure and function were damaged. However, when we overexpressed mtCx43 in Rictor-knockdown cells, mitochondrial dysfunction and the changes in the cardiac differentiation rate were ameliorated, which indicated that Rictor modulates mitochondrial function via mtCx43. Furthermore, it was reported that the expression of mtCx43 is modulated by Akt activation. The expression of mtCx43 in the rat cortex was reduced by treatment with the PI3K/Akt pathway inhibitor LY 294002 (LY) [35].

**Fig. 5:**
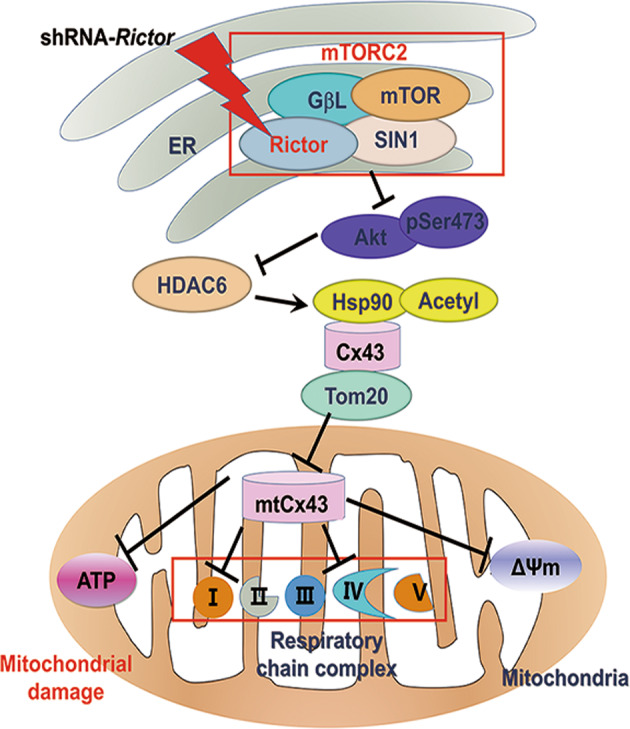
Schematic representation of the mechanisms by which Rictor regulates mitochondrial function in ESC-CMs.

Cx43 transport to the mitochondrial inner membrane is dependent on the Hsp90-TOM20 transport system [30]. In addition, the deacetylation of Hsp90, mediated by HDAC6, has been shown to be involved in the mitochondrial transport of proteins [36, 37]. In addition, HDAC6 inactivation and knockdown experiments showed that HDAC6 contributes to Hsp90 hyperacetylation and the loss of Hsp90 chaperone activity [38]. Meanwhile, it was revealed that in head and neck squamous cell carcinoma, inhibition of mTOR/Akt pathway signalling inhibits HDAC6 [39, 40]. Consistently, in our study, we found that knockdown of Rictor resulted in inactivation of the mTOR/Akt pathway and subsequently decreased HDAC6 expression in ES-CMs. As a result, in ESC-CMs, the increased acetylation of Hsp90 mediated by HDAC6 inhibition disturbed Cx43 translocation. These results suggested that knockdown of Rictor could interfere with the translocation of Cx43 from the plasma to mitochondria by decreasing HDAC6 expression. In conclusion, knockdown of Rictor resulted in mitochondrial dysfunction owing to the inhibition of Cx43 translocation to mitochondria. Mechanistically, this effect might have involved inactivation of the mTOR/Akt signalling pathway and a subsequent decrease in Hsp90-Cx43-TOM20 formation attributed to Hsp90 hyperacetylation mediated by HDAC6 inhibition due to Rictor knockdown. Therefore, mtCx43 participated in shRNA-*Rictor*-induced mitochondrial function damage in ESC-CMs, providing an experimental basis for further investigation of the mechanisms of Rictor-regulated cardiomyocyte differentiation (Fig. 5).

## Supplementary information

supplemental Fig. 1
