# Supplementary figures and images for: Rictor/mTORC2 involves mitochondrial function in ES cells derived cardiomyocytes via mitochondrial Connexin 43
Source: Acta Pharmacol Sin. 2021 Feb 5;42(11):1790–7. doi: 10.1038/s41401-020-00591-3 (PMC8563760; doi:10.1038/s41401-020-00591-3)

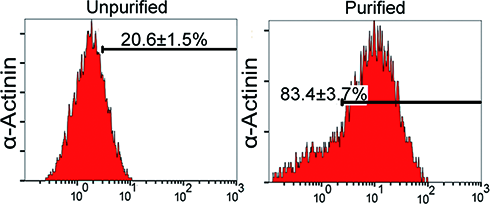

Supplement: Supplementary file 1 — supplemental Fig. 1 [file 41401_2020_591_MOESM1_ESM.tif]
